# Contemporary office-based procedures in rhinology: a narrative review of techniques, indications, and outcomes

**DOI:** 10.3389/falgy.2025.1554792

**Published:** 2025-07-18

**Authors:** Ghassan Alokby

**Affiliations:** ^1^ Department of Otolaryngology - Head and Neck Surgery, King Faisal Specialist Hospital and Research Center, Riyadh, Saudi Arabia; ^2^ College of Medicine, Alfaisal University, Riyadh, Saudi Arabia

**Keywords:** office-based rhinology, balloon sinuplasty, cryotherapy, radiofrequency neurolysis, nasal valve collapse, eustachian tube dilation office-based rhinology, minimally invasive procedures, turbinate reduction

## Abstract

**Background:**

The role of office-based procedures in rhinology has expanded considerably, driven by advancements in minimally invasive techniques and a growing emphasis on value-based care. These interventions can offer effective management for selected sinonasal conditions while reducing reliance on operating room resources.

**Objective:**

To provide a comprehensive narrative review of contemporary office-based rhinologic procedures, focusing on indications, anesthetic considerations, patient selection, and safety protocols. Additionally, to share institutional experience, highlighting practical benefits in workflow optimization and patient access.

**Methods:**

A narrative review was conducted by searching PubMed, Embase, and Scopus databases for English-language articles published between [2010–2025] using the keywords: *office-based rhinology*, *balloon sinuplasty*, *cryotherapy*, *radiofrequency neurolysis*, *nasal valve collapse*, and *Eustachian tube dilation*. Priority was given to clinical studies, systematic reviews, and guidelines relevant to procedural safety, efficacy, and healthcare resource utilization. Additionally, institutional data from King Faisal Specialist Hospital and Research Centre (KFSHRC) were reviewed to illustrate real-world application.

**Results:**

The review highlights key office-based procedures, their indications, contraindications, anesthetic strategies, and safety considerations. Institutional experience demonstrated a 140% increase in office-based procedures over two years, reduced waiting times, and improved operating room efficiency, particularly for routine turbinate surgeries.

**Conclusion:**

Office-based rhinologic procedures offer safe, effective, and resource-efficient alternatives for selected patients. While current evidence and institutional experiences are promising, further research is warranted to standardize practice, evaluate long-term outcomes, and assess economic impacts across healthcare systems.

## Introduction

Office-based rhinologic procedures have undergone remarkable advancements over the past decade. In 2014, Ruiz et al. provided one of the earliest structured overviews of office-based rhinologic procedures, focusing primarily on their potential to deliver minimally invasive care for sinonasal conditions, with limited techniques available at the time, such as sinonasal debridement and balloon sinuplasty (1). Since then, the field has expanded rapidly, fueled by the development of novel technologies including cryotherapy, radiofrequency neurolysis (e.g., Vivaer), bioabsorbable nasal valve implants (e.g., Latera), and balloon dilation for both sinuses and the Eustachian tube.

An emerging body of evidence suggests that, in selected populations, these procedures may offer benefits in terms of safety, symptom relief, and patient satisfaction  (2–4). Their growing use reflects a gradual shift toward minimally invasive, outpatient care that aligns with value-based healthcare principles.

The clinical indications for office-based rhinology have also broadened. While early applications focused on simple interventions like polypectomy or turbinate reduction (1), contemporary practices now address a wide array of conditions including chronic rhinitis, nasal valve collapse, Eustachian tube dysfunction, and mild to moderate chronic rhinosinusitis. For example, cryotherapy has emerged as a treatment option for chronic rhinitis refractory to pharmacologic management, while balloon sinuplasty offers a less invasive alternative for patients with sinus ostial obstruction  (5).

In selected cases, these interventions may reduce reliance on operating rooms and general anesthesia, potentially leading to shorter recovery times and improved access to care. However, the clinical applicability and institutional adoption of these procedures may vary depending on available resources, procedural costs, and provider expertise.

This review explores the evolution and current role of office-based procedures in rhinology, categorizing interventions into functional and structural domains. We also describe our institutional experience at King Faisal Specialist Hospital and Research Centre (KFSHRC), where selected in-office procedures have been integrated into routine practice. The review emphasizes safety, patient selection, anesthetic considerations, and potential limitations, with the goal of guiding informed implementation in similar clinical settings.

## Search methodology

A narrative review was conducted by searching PubMed, Embase, and Scopus databases for English-language articles published between [2010–2025] using the keywords: *office-based rhinology*, *balloon sinuplasty*, *cryotherapy*, *radiofrequency neurolysis*, *nasal valve collapse*, and *Eustachian tube dilation*. Priority was given to clinical studies, systematic reviews, and guidelines relevant to procedural safety, efficacy, and healthcare resource utilization. Additionally, institutional data from King Faisal Specialist Hospital and Research Centre (KFSHRC) were reviewed to illustrate real-world application.

## Functional interventions

Office-based functional interventions address symptoms of chronic rhinitis and related conditions with minimally invasive techniques. Among these, cryotherapy and radiofrequency neurolysis are new novel techniques to manage these symptoms, especially when refractory to the appropriate medical treatment.

## Cryotherapy

Chronic rhinitis is a debilitating condition characterized by persistent nasal symptoms, including rhinorrhea, nasal congestion, sneezing, and nasal itching, which can significantly impair quality of life (6). Management options range from medical therapies, such as antihistamines, intranasal steroids, and anticholinergic sprays, to more invasive procedures like vidian or posterior nasal neurectomy in refractory cases (7).

Cryotherapy offers a minimally invasive alternative by using targeted cold-induced ablation of the posterior nasal nerve (PNN) within the posterior lateral nasal wall. This technique preserves surrounding structures while aiming to disrupt parasympathetic innervation to reduce rhinorrhea and congestion. A commercially available device, ClariFix™, delivers nitrous oxide cryogen via an endoscopically guided probe to achieve this ablation. The technology was approved for use in the U.S. in 2016 and in Canada in 2021 (7, 8).

A systematic review of eight studies, including one randomized controlled trial and seven prospective cohort studies, evaluated outcomes in 472 patients undergoing cryotherapy for chronic rhinitis (8). Symptom improvement was consistently demonstrated using validated tools such as the Total Nasal Symptom Score (TNSS) and the reflective TNSS (rTNSS), with follow-up durations extending up to 24 months. These findings suggest sustained symptom relief over time in many patients.

In the same review, Patient-reported outcomes further underscored the efficacy of cryotherapy. Improvements in the Runny Nose Score (RNS) from the 22-item Sino-Nasal Outcomes Test (SNOT-22). Mean pre-procedural SNOT-22 RNS scores of 4.2 were reduced by >1 point in 71% of patients following the procedure. The most commonly reported adverse event was post-procedure pain, occurring in 13.5% of cases (64 patients). Headache was reported in 4.23% (20 patients), followed by numbness in 2.96% (14 patients). Less frequent complications included nasal congestion or sinusitis in 1.48% (7 patients), bleeding in 1.06% (5 patients), and watery eyes in 0.64% (3 patients). Overall, ClariFix™ demonstrates a relatively low complication rate, with most adverse events being mild and self-limited (8).

A more recent multicenter study by Craig et al.  further explored the durability of cryotherapy in patients with rhinitis unresponsive to ipratropium bromide. Out of 74 patients treated with cryoablation, 84% experienced initial symptom improvement. However, among the 60 patients with adequate long-term follow-up (mean 31.6 months), 95% experienced some degree of symptom recurrence at a mean of 5 months post-treatment. Notably, 65% recurred to baseline severity, and 90% of those with recurrence expressed interest in further treatment. These results suggest that while cryotherapy is effective in the short term, the durability of benefit may be limited in patients with prominent secretory symptoms (9).

## Radiofrequency neurolysis

Radiofrequency neurolysis is a minimally invasive, office-based treatment for chronic rhinitis that targets the posterior nasal nerve (PNN). The RhinAer™ device delivers temperature-controlled radiofrequency (RF) energy to the posterior lateral nasal wall, aiming to interrupt parasympathetic nerve signaling responsible for rhinorrhea and nasal congestion. This is typically performed under local anesthesia in an outpatient setting (10).

Yu et al. conducted a systematic review and meta-analysis including over 200 patients treated with RF neurolysis, reporting significant and sustained improvements in the Total Nasal Symptom Score (TNSS) and reflective TNSS (rTNSS) across follow-up periods ranging from 3–12 months (11). Similarly, Lee et al. demonstrated meaningful reductions in rhinologic symptoms and overall quality-of-life improvements after treatment with the RhinAer™ device  (10). These studies concluded that RF neurolysis is generally well-tolerated, with a favorable safety profile. Reported side effects are typically mild and include transient nasal dryness or local discomfort (10, 11).

## Comparison of cryotherapy, radiofrequency neurolysis, and surgical neurectomy

A recent comparative study by Maddineni et al. evaluated outcomes of in-office PNN ablative procedures (cryotherapy and radiofrequency neurolysis) against surgical neurectomy in patients with chronic rhinitis refractory to medical therapy. All three interventions demonstrated improvements in rhinologic symptoms, but the study highlighted symptom-specific differences. Surgical neurectomy was associated with statistically significant improvements in sneezing, rhinorrhea, and postnasal drip, while in-office ablation techniques showed modest benefits and, in the case of radiofrequency neurolysis, a potential worsening of sneezing scores. Additionally, only the neurectomy group achieved minimal clinically important difference (MCID) in rhinologic subdomain SNOT-22 scores (12).

Despite these findings, in-office interventions remain preferable in many clinical contexts due to their minimally invasive nature, low risk profile, and feasibility under local anesthesia. They are particularly suitable for patients who are poor candidates for general anesthesia, have multiple comorbidities, or prioritize rapid recovery and convenience. These considerations reinforce the importance of individualized treatment selection based on symptom burden, procedural tolerance, and patient preference.

## Structural interventions

Structural interventions in office-based rhinology address anatomical issues contributing to nasal obstruction, offering minimally invasive solutions for conditions such as nasal valve collapse and inferior turbinate hypertrophy. Key procedures include the use of bioabsorbable implants for nasal valve repair advanced techniques for turbinate reduction and balloon dilation of the sinus ostia or the Eustachian tube.

## Nasal valve treatment with latera implants

Internal and external nasal valve collapse, collectively termed lateral wall insufficiency (LWI), has been identified as a significant cause of nasal obstructive symptoms. The American Academy of Otolaryngology–Head and Neck Surgery recently issued a clinical statement recognizing LWI as a distinct clinical entity impacting nasal airflow (13). Traditional surgical interventions, such as rhinoplasty, have been the mainstay of treatment for LWI. However, less invasive options, like the Latera™ bioabsorbable implant, have gained prominence in office-based settings (3).

Kim et al. performed a meta-analysis including five studies with 396 participants to evaluate the outcomes of bioabsorbable nasal implants for LWI. The analyzed outcomes included disease-specific quality of life (QOL) measures such as the Nasal Obstruction Symptom Evaluation (NOSE) and visual analogue scale (VAS), as well as endoscopic scores. Adverse effects, including implant retrieval, pain, foreign body sensation, localized swelling, and mucosal infection, were also assessed.

The meta-analysis concluded that bioabsorbable nasal implants significantly improved QOL scores and reduced lateral wall motion on endoscopy compared to baseline. These benefits were sustained for up to one year postoperatively. Most adverse effects were mild, transient, and resolved without significant sequelae. Compared to sham surgeries, nasal implants demonstrated a superior ability to improve disease-specific QOL, making them a valuable addition to LWI management (3).

This approach reduces the need for general anesthesia and lengthy recovery periods, aligning well with modern, minimally invasive care paradigms; however, long-term data remains limited and further studies are needed to understand the sustainability of the improvement.

## Inferior turbinate reduction

Inferior turbinate hypertrophy is a known contributor to nasal obstruction and often coexists with chronic rhinitis (14). While conventional surgical techniques such as partial turbinectomy or submucosal resection remain widely used, several office-based alternatives have emerged, including radiofrequency ablation and coblation-assisted turbinoplasty. These techniques aim to reduce turbinate volume while preserving mucosal integrity, allowing for symptomatic relief with minimal tissue trauma (14).

Coblation technology employs plasma-mediated ablation to reduce turbinate volume by vaporizing soft tissue with minimal thermal injury to surrounding structures (14). In a prospective non-randomised trial by Di Rienzo Businco et al. the researchers evaluated coblation turbinoplasty in 220 patients, with 110 receiving surgery followed by medical therapy and 110 receiving medical therapy alone. The efficacy of the treatment was measured by evaluating subjective nasal symptoms, rhino manometric values after specific nasal provocation tests (NPTs), and rhinoendoscopy. The researchers concluded that coblation-assisted inferior turbinoplasty, when combined with medical therapy, has been shown to improve nasal airflow more effectively than medical treatment alone in patients with persistent moderate to severe allergic rhinitis. Notably, local nasal reactivity, as assessed by NPT, demonstrated a significant reduction (14).

Similarly, radiofrequency turbinoplasty uses controlled thermal energy to shrink submucosal tissues. Comparative analyses have shown that radiofrequency achieves symptom relief similar to coblation, with high rates of patient satisfaction and minimal recovery times (15).

A meta-analysis reviewed the outcomes of radiofrequency ablation (RF) and microdebrider-assisted turbinoplasty (MAT) in addressing bilateral inferior turbinate hypertrophy. Both techniques showed significant improvements in subjective nasal obstruction as measured by the Visual Analog Scale (VAS), and in objective parameters like nasal airflow, volume, and resistance assessed through acoustic rhinomanometry. While RF demonstrated equivalent short-term efficacy to MAT, the two highest-quality studies favored MAT for long-term outcomes. The review highlighted the safety and effectiveness of RF techniques under local anesthesia, with minimal complications reported. However, limitations included substantial heterogeneity across studies, reliance on short-term data, and variability in RF device settings and patient populations. This underscores the need for standardized methodologies and long-term follow-up in future research (16).

## Nasal valve remodeling

Nasal valve collapse (NVC) is a major contributor to nasal obstruction, often necessitating intervention to restore airflow and alleviate symptoms (13). The Vivaer™ device, developed by Aerin Medical, utilizes temperature-controlled radiofrequency energy to remodel the nasal valve, stiffening the lateral nasal wall with the aim of improving airflow without altering the external nasal appearance (17).

Casale et al. conducted a systematic review and meta-analysis to evaluate the efficacy of the Vivaer device (4). Four studies, including a total of 297 patients, were analyzed from 5 studies. These studies utilized the Nasal Obstruction Symptom Evaluation (NOSE) score as the primary outcome measure to assess patient-reported improvements in nasal obstruction severity. The pooled data demonstrated a significant reduction in NOSE scores, with a mean difference of 46.13 points three months after treatment (95% CI, 43.27–48.99). This improvement exceeded the minimum clinically important difference for the NOSE score, indicating meaningful symptomatic relief.

Advers events were minimal across the included studies, with reported issues limited to transient nasal congestion, swelling, and mild pain during the first month post-procedure. Importantly, no changes in external nasal appearance were observed, underscoring the cosmetic safety of the treatment and all adverse effects resolved during the follow up period of the study.

The meta-analysis highlighted moderate heterogeneity among the studies, attributed to differences in study designs and patient populations. Despite this, the findings suggest the Vivaer procedure's safety, efficacy, and applicability in an office-based setting under local anesthesia (4).

## Balloon dilation of the eustachian tube (BDET)

Balloon dilation of the Eustachian tube (BDET) is a minimally invasive procedure designed to address Eustachian tube dysfunction (ETD), particularly the dilatory subtype, which is characterized by the inability of the tube to open adequately, leading to negative middle ear pressure and associated symptoms such as aural fullness, hearing loss, and tinnitus. When severe, patients may experience otalgia and may develop serous otitis media and complications such as atelectasis, retraction pockets, or even cholesteatoma (18).

Proper patient selection is importing for a successful outcome. The ideal candidates typically have symptoms lasting over 12 weeks, persistent aural fullness or barometric sensitivity, type B or C tympanograms, and Eustachian Tube Dysfunction Questionnaire (ETDQ-7) scores greater than 14  (6). Patients with patulous ETD, ossicular chain pathology, or normal tympanograms are generally not suitable for the procedure (19).

Outcomes from randomized controlled trials by Meyer et al. demonstrate significant improvements in ETDQ-7 scores and tympanogram normalization following BDET, with success rates ranging from 64%–97%. The study reported that treated patients experienced normalization of tympanograms (51.8% vs. 13.9% in untreated patients) and clinically meaningful reductions in ETDQ-7 scores (56.2% vs. 8.5% in untreated patients) at 6-week follow-up. These improvements were durable, with sustained benefits observed over one year (20). In a systematic review involving 1,155 patients, multiple assessment modalities—including ETDQ-7 scores, Valsalva maneuver/Toynbee test, tympanometry, and audiometry—consistently demonstrated both short-term and long-term improvement following balloon Eustachian tube dilation, with an average follow-up duration of 6.9 months (21).

Balloon dilation of the Eustachian tube is generally considered safe. However, a recent analysis of the FDA's Manufacturer and User Facility Device Experience (MAUDE) database, covering reports from January 2000 to July 2022, identified several adverse events associated with this intervention. The most frequently reported complication was subcutaneous emphysema, occurring in 8 out of 13 documented cases, with some instances requiring hospitalization or antibiotic therapy. Other less common adverse events included patulous Eustachian tube (*n* = 2), vascular dissection leading to stroke (*n* = 1), nasopharyngeal mucocele (*n* = 1), and tinnitus (*n* = 1). While most patients recovered fully, two individuals experienced persistent symptoms post-procedure (22). These findings underscore the importance of thorough patient counseling regarding potential risks, even though such complications are rare as well as proper evaluation of the patient including having a pre-procedure high resolution CT scan to evaluate for any internal carotid Artery dehiscence.

## Balloon sinuplasty

Balloon sinuplasty is used to treat selected cases of chronic rhinosinusitis without nasal polyposis (CRSsNP) and recurrent acute rhinosinusitis (RARS). The technique involves endoscopic placement of a balloon catheter into the sinus ostium. Upon inflation, the balloon widens the ostium through microfracturing of adjacent bony structures, preserving the mucosa and restoring physiologic sinus drainage (23).

The procedure is generally considered for patients with isolated maxillary, frontal, or sphenoid sinus involvement, particularly when imaging confirms anatomical obstruction. It is not appropriate for those with diffuse ethmoidal disease, polyposis, invasive fungal sinusitis, or radiologically normal sinus cavities  (24).

A prospective, multicenter study by Bolger et al. evaluated the safety and outcomes of balloon catheter sinusotomy in 115 patients and 307 sinus ostia over a 24-week period  (25). Endoscopic evaluation at study conclusion demonstrated that 80.5% of all treated sinuses (247/307) were patent. Among those where ostial status could be determined endoscopically (252/307), 98% were patent. The procedure was well tolerated, with a low revision rate of 0.98% per sinus and 2.75% per patient. Symptomatically, patients experienced consistent improvement in Sino-Nasal Outcome Test (SNOT-20) scores over baseline, indicating a favorable impact on quality of life. Adverse events were rare and mild in severity.

Weiss et al. evaluated 65 patients (195 ballooned sinuses) two years post-procedure (26). This cohort included both “balloon-only” and “hybrid” (balloon + other techniques) cases. Across the entire group, SNOT-20 scores improved significantly (from 2.17 at baseline to 0.87 at two years, *P* < 0.001), and scores remained stable compared to six-month and one-year follow-up. Both subgroups, balloon-only and hybrid, showed similar levels of improvement. In parallel, Lund-Mackay CT scores improved from 9.66–2.69 overall, again with consistent improvement in both subsets. Eighty-five percent of patients reported symptomatic improvement, with no patients reporting worsening. Revision was required in 7 of 195 sinuses (3.6%), affecting 6 of 65 patients (9.2%).

Taken together, these data support the short- and intermediate-term efficacy of balloon sinuplasty in appropriately selected cases.

Despite its minimally invasive nature and overall safety profile, balloon sinuplasty is not without risks. A nationally representative retrospective cohort study of over 16,000 patients found a 5.26% complication rate among those undergoing balloon sinuplasty, compared to 7.35% for conventional FESS. The revision surgery rate for BSP was 7.89%, which, while lower than that for FESS (16.85%) and hybrid procedures (15.15%), still underscores the need for patient-specific risk assessment. Although serious complications are rare, reported events included cerebrospinal fluid leaks, pneumocephalus, orbital injuries, and severe bleeding (23). These risks should be clearly discussed with patients during surgical counseling. Further prospective studies are needed to directly compare the long-term efficacy and safety of BSP vs. conventional endoscopic approaches.

## In-office endoscopic Sinus surgery

In recent years, more invasive office-based procedures such as functional endoscopic sinus surgery (ESS) have gained popularity, but the evidence regarding their safety remains limited (27). However, one of the largest published series on complete ESS in the office setting appeared in 2017. In that study, Scott JR and colleagues reviewed 118 patients who underwent comprehensive ESS on 196 sinus sides. These surgeries involved opening obstructed sinus pathways and were more extensive than simple polypectomies. The average follow-up was 13.4 months. During the follow-up period, nine patients (7.6%) required revision surgery. Of these, eight underwent additional procedures under general anesthesia in the operating room, while one had a repeat procedure in the clinic. In seven cases (5.9%), the initial surgery had to be stopped early due to pain, bleeding, or vasovagal reactions. Four of these cases (3.3%) involved intraoperative pain that could not be controlled with additional local anesthesia, necessitating early termination and rescheduling under general anesthesia (28).

More recently, Kokavec et al. conducted an eight-year retrospective study evaluating the safety of in-office rhinology procedures, including ESS. They found that 2.5% (8 out of 314) of procedures were prematurely terminated due to vasovagal or syncopal episodes and/or bleeding. Postoperative complications—such as bleeding, infection, or significant pain—were reported in 5.4% of cases (17 out of 314). The revision surgery rate for ESS in their cohort was 10% (32 out of 314), with an average follow-up duration of 15.8 months (29).

## Anesthesia considerations in office-based rhinology

Anesthesia plays a critical role in ensuring the success and safety of office-based rhinology procedures. Various studies highlight the importance of tailored anesthesia protocols to optimize patient comfort while minimizing risks. Techniques commonly employed include local anesthesia with or without mild sedation, allowing for effective analgesia and rapid recovery (30–33).

For procedures such as balloon sinuplasty, cryotherapy, and radiofrequency neurolysis, topical anesthetics, typically lidocaine spray combined with epinephrine for hemostasis, are widely used. Local infiltration of lidocaine or bupivacaine provides additional analgesia, particularly in more invasive interventions such as turbinate reduction or nasal valve remodeling (3, 4, 15, 25). In balloon dilation of the Eustachian tube, Dean and Pynnonen emphasize the utility of transnasal topical anesthetics to effectively numb the mucosa. In some cases, oral sedatives or intranasal dexmedetomidine may be administered to reduce anxiety and enhance patient cooperation. Premedication with a vestibular suppressant such as 10 mg of diazepam 90 minutes before the procedure has also been recommended to limit vertigo related to barometric pressure changes during Eustachian tube dilation  (19).

While local anesthesia avoids the systemic risks associated with general anesthesia, appropriate patient selection remains foundational to procedural safety. Ideal candidates are alert, cooperative, and able to tolerate nasal instrumentation without distress. However, patients with active infections, cardiovascular disease, coagulopathy, insulin-dependent diabetes, obesity, obstructive sleep apnea, poorly controlled hypertension, or thromboembolic disease are generally considered poor candidates for office-based procedures. Additional relative contraindications include severe anxiety, a history of vasovagal syncope, or the inability to remain supine for the duration of the intervention (30, 31).

In addition to patient-related considerations, the procedural setting must be equipped to support safe anesthetic administration. Offices should be outfitted with monitoring equipment, resuscitation supplies, and emergency medications, including oxygen, epinephrine, antihistamines, and reversal agents. The presence of trained personnel is essential for any procedure involving anxiolytics or deeper sedation. Furthermore, institutions should have clearly defined protocols for stabilizing patients and for transferring them promptly to higher levels of care in the event of a complication (30, 32, 34).

Although adverse events are uncommon, clinicians should remain vigilant for vasovagal reactions, local tissue responses, and rarely, arrhythmias or bronchospasm (35). Thorough pre-procedural counseling and a clear plan for managing intolerance or aborted procedures are essential components of safe office-based practice (30).

## Discussion

Office-based rhinology has evolved significantly over the past decade, with a growing number of therapeutic procedures now routinely performed outside the operating room. A survey conducted among members of the American Rhinologic Society (ARS) found that 99% of respondents perform office-based rhinologic procedures, with sinonasal debridement (99%), polypectomy (77%), and balloon sinus ostial dilation (56%) being the most commonly performed. Respondents also reported increasing use of advanced technologies in the office setting, including steroid-eluting implants and computer-assisted navigation. Notably, 63% of participants indicating an increased adoption of office-based, minimally invasive procedures among rhinologists (10).

When applied to appropriately selected patients, office-based procedures may reduce the burden on operating rooms, improve scheduling efficiency, and offer more convenient care pathways. From a cost perspective, these interventions can offer meaningful savings in several areas. Eliminating the need for hospital operating rooms significantly reduces facility-related charges, while the use of local rather than general anesthesia lowers anesthetic costs. Additionally, shorter recovery times allow many patients to resume daily activities or return to work sooner, thereby reducing indirect costs and minimizing productivity loss. However, due to variability in reimbursement structures and case complexity, these benefits are context-dependent and should be evaluated at the institutional level. Broad claims regarding cost-effectiveness require further health economic validation through prospective studies (2, 5, 36, 37).

At King Faisal Specialist Hospital and Research Centre (KFSHRC), the integration of office-based rhinologic procedures has helped address institutional challenges related to OR congestion and patient flow. Given the hospital's dual role as a national tertiary care center and provider of primary and secondary care to its employee population, surgical prioritization has historically favored complex cases such as malignancies and skull base pathology. As a result, a significant waiting list developed for patients with routine conditions that could be managed more efficiently in an office setting.

To address this, a structured office-based rhinology program was implemented. Although not yet as extensive as some international counterparts, the initiative has led to reductions in elective case backlog, improved OR availability, and enhanced patient satisfaction. Investments included the establishment of procedure-equipped clinic rooms, designated scheduling slots, and support from specialized nursing staff. These changes have optimized workflows and allowed for the expansion of services within a resource-conscious framework. This lead to a substantial growth in in-office procedures in recent years. An analysis of cases performed by the author in 2023 and 2024 demonstrated a 140% increase in office-based interventions, with 48 cases completed in 2024. The majority of these procedures involved inferior turbinate coblation, alongside other interventions such as bioabsorbable nasal valve implants, cryotherapy, balloon dilation of the Eustachian tube, balloon sinuplasty, and PRP injections.

This strategic shift has contributed to more efficient surgical resource allocation. While total OR utilization increased in 2024 due to a higher volume of complex tertiary cases, routine procedures particularly pure turbinate surgeries were transitioned to the office setting. The number of cases booked in the operating room only for turbinate surgeries dropped from 12 cases in 2023 to just 1 case in 2024, freeing operating room capacity for advanced oncologic and skull base surgeries.

In addition to optimizing OR usage, this transition significantly reduced patient waiting times. Previously, patients requiring turbinate reduction often faced delays of several months before being scheduled for surgery in the OR due to prioritization of more complex cases. By diverting eligible patients to office-based procedures, scheduling times were reduced to typically 1–2 weeks, enhancing patient access to care and improving satisfaction. These outcomes align with a recent scoping review published in the *journal of the American Academy of Otolaryngology-Head and Neck Surgery*, which identified de-escalation of care levels as a key strategy to improve surgical efficiency. Approaches such as shifting procedures to minor settings, utilizing local anesthesia, and reserving OR capacity for high-acuity cases were highlighted as effective methods (5).

A unique aspect of the KFSHRC experience is its high proportion of medically complex patients, including those with cystic fibrosis, post-transplant status, and other chronic illnesses. For these individuals, general anesthesia may carry substantial risk. In such cases, the availability of in-office procedures such as balloon sinuplasty, limited polypectomy, and turbinate coblation, has provided a safe and effective alternative that minimizes perioperative morbidity while maintaining therapeutic benefit.

Despite their advantages, office-based procedures are not suitable for all patients. Careful selection is critical. Patients with extensive sinonasal disease, significant anatomical distortion, or indications for tissue removal beyond the reach of local anesthesia are more appropriately managed in the operating room (1, 2, 23). Similarly, individuals with poorly controlled anxiety, inability to tolerate nasal instrumentation, or medical comorbidities requiring close anesthetic monitoring may be excluded from in-office procedures (30, 31).

Relative contraindications include active infection, coagulopathy, uncontrolled hypertension, obstructive sleep apnea, obesity, thromboembolic disease, and advanced cardiovascular conditions. Pediatric patients and individuals with a history of vasovagal syncope or intolerance to endoscopy may also be unsuitable. Nonetheless, some patients who are too high-risk for general anesthesia may still benefit from carefully selected, low-risk office-based interventions (33).

Pre-procedural counseling is a cornerstone of successful implementation. Patients should be fully informed about the risks, benefits, and alternatives of office-based procedures, and actively involved in the decision-making process. This collaborative approach improves satisfaction, enhances safety, and supports shared accountability.

## Conclusion

Office-based rhinologic procedures represent a significant advancement in the management of selected sinonasal conditions, offering minimally invasive alternatives to traditional surgical approaches. Supported by growing clinical evidence, these interventions have demonstrated favorable safety profiles, high patient satisfaction, and the potential to optimize healthcare resource utilization when applied in appropriate clinical contexts. However, the successful implementation of office-based procedures depends on rigorous patient selection, adherence to standardized anesthetic and safety protocols, and institutional readiness, including proper equipment and trained personnel. While short- and intermediate-term outcomes are promising, further research is needed to evaluate long-term efficacy, cost implications, and direct comparisons with operating room-based interventions.

As technology and clinical experience continue to evolve, office-based rhinology is poised to play an increasingly important role in delivering patient-centered, efficient care. Ongoing efforts to refine patient selection criteria, expand procedural capabilities, and establish evidence-based guidelines will be essential in ensuring that these advances translate into sustainable improvements in outcomes and healthcare value.
